# Geriatric nursing career motivation among Chinese undergraduate nursing students: the mediation role of geriatric nursing competence

**DOI:** 10.1186/s12912-026-04593-0

**Published:** 2026-04-02

**Authors:** Yifan Chen, Ting Yi, Yiwen Hu, Ziling Xie, Yan Wang, Yi Qi, Chaoqun Dong

**Affiliations:** 1 School of Nursing, Wenzhou Medical University, University Town, Chashan, Wenzhou, Zhejiang 325035 China; 2 Cixi Biomedical Research Institute, Wenzhou Medical University, Zhejiang, China

**Keywords:** Geriatric nursing, Career motivation, Competence, Attitudes, Older people, Mediating effect

## Abstract

**Background:**

The unprecedented demographic shift toward an aging global population poses substantial challenges to healthcare systems worldwide, particularly in China. Despite the increasing demand for specialized geriatric nursing, a trend has emerged in which nursing students consistently demonstrate a reluctance toward pursuing careers in geriatric nursing, resulting in a critical shortage of qualified healthcare professionals in this essential field.

**Objectives:**

This study aimed to examine the interrelationships among nursing students’ attitudes toward older people, their geriatric nursing competence, and their motivation to pursue careers in geriatric nursing; and to investigate the potential mediating role of geriatric nursing competence in the relationship between attitudes toward older people and geriatric nursing career motivation.

**Methods:**

A cross-sectional survey was conducted using a convenience sample at Wenzhou Medical University from June 2023 to August 2023. Data were collected using the Chinese version of the Kogan’s Attitudes toward Old People (KAOP) scale, Geriatric Nursing Career Motivation Questionnaire, and Geriatric Nursing Competence Scale for Clinical Nurses which used an online questionnaire. Structural equation modeling (SEM) was used to test the hypothesized mediating role of geriatric nursing competence.

**Results:**

This study recruited 529 nursing students across various academic years. Pearson analysis revealed a positive correlation between attitudes toward older people and geriatric nursing competence (*r* = 0.106, *p* < 0.05), as well as between attitudes toward older people and geriatric nursing career motivation (*r* = 0.375, *p* < 0.01). SEM revealed that attitudes toward older people had a significant direct effect on the motivation to choose a career in geriatric nursing (direct effect = 0.146, 95% CI [0.028–0.296]), with geriatric nursing competence partially mediating this relationship (indirect effect = 0.028, 95% CI [0.009–0.050], accounting for 16.09% of the total effect).

**Conclusions:**

Attitudes toward older people had primarily direct effect on geriatric nursing careers motivation, with a modest indirect effect mediated by geriatric nursing competence. Educational initiatives should focus on fostering positive attitudes toward older people while complementarily enhancing students’ geriatric nursing competence to address the growing need for geriatric nursing professionals.

**Clinical trial number:**

Not applicable.

## Introduction

The global demographic shift toward an aging population presents unprecedented challenges to healthcare systems worldwide. The World Health Organization predicts that the proportion of the world’s population aged 60 years and above will double between 2015 and 2050, from 12% to 22% [1]. The situation in China is even more serious, the proportion of the population aged 65 years reached 14.2% in 2021, rising to 14.9% by 2022 [2]. According to conservative estimates, China may cross the threshold of a super-aging society by 2032, when the population aged 65 years and above will account for more than 20% of the total population [2]. This rapid demographic shift reflects China’s profound societal transformation and emerging challenges in the economic, social, and public service sectors [3]. However, despite the divergence in national approaches to the increasing aging population, a universally trained professional nursing workforce remains a critical strategic priority [4]. Yet current global systems are insufficient to meet demand [5], especially in low- and middle-income countries [6]. This crisis has been further intensified by worldwide workforce shortages [7], highlighting the urgent needs to cultivate professional talent. In this context, nursing students are gradually becoming an important talent pool in the future geriatric nursing system [8, 9]. However, the current landscape remains discouraging, as many nursing students perceive geriatric nursing career as a challenging and unappealing profession [3, 8, 10]. Research across six European countries found low interest in geriatric nursing career among graduating nursing students [10]. Consequently, nursing education must actively foster the geriatric nursing career motivation among students.

Numerous studies have investigated the factors influencing nursing students’ geriatric nursing career motivation, including personal, educational, and societal elements. Key determinants include attitudes toward older people [3, 11–13], geriatric nurse teachers’ ability [14], knowledge and skills [15], aging anxiety [3, 11, 13], the clinical practice environment [3, 11], empathy [3], gratitude [16], and experience in nursing care for older people [12, 13, 15]. Notably, attitudes toward older people are associated with students’ willingness to work in geriatric nursing career. A longitudinal research emphasizes the importance for cultivating students’ positive attitudes, empathy, and reducing anxiety about aging in nursing education [3]. Cross-cultural studies in China [9] and Turkey [15] have consistently shown that positive attitudes toward older people might play a key role in shaping nursing students’ geriatric nursing career motivation. These attitudes, which may be shaped by personal experiences, cultural norms, and societal perceptions of aging, are associated with the quality of care provided to older people [17]. Kogan’s [18] seminal work on attitudes toward older people laid the foundation for understanding how these perceptions affected interactions with and care for older people. Subsequent studies have consistently demonstrated that positive attitudes toward older people are associated with higher quality care and better health outcomes for older patients [8].

Research indicates that students were reluctant to pursue a career in geriatric nursing because of their lack of knowledge and skills [15]. Geriatric nursing competence, the cornerstone of effective care for older people, encompasses a wide range of specialized skills and abilities. In 2018, the American Nurses Association issued standards outlining competencies for geriatric nurses across assessment, diagnosis, outcome identification, planning, implementation, and evaluation [19]. This competence emphasizes evidence-based, person-centered care across various settings, which is essential for navigating the current healthcare landscape. Studies have demonstrated that well-prepared nurses provide significantly higher-quality care, leading to enhanced patient outcomes [20]. Ultimately, the development and application of geriatric nursing competence are intrinsically linked to attitudes toward older people and geriatric nursing career motivation [21].

The relationship between nursing students’ attitudes toward older people and their geriatric nursing competence remains understudied, with only a few studies reporting an association between these two aspects. Studies have shown a positive correlation between attitudes toward older people and geriatric nursing competence in nursing students [22] and hospital nurses [23]. Additional studies have approached this relationship from a different perspective, showing that positive attitudes and the desire to interact with older people are correlated with the level of care provided [24]. The concepts and attitudes of medical staff directly determine the level of care received by older patients and affect their quality of life and medical experience [25]. However, geriatric nursing competence remains the fundamental basis for ensuring quality of care. Therefore, the direct relationship between attitudes toward older people and their geriatric nursing competence among nursing students should be demonstrated.

Understanding what motivates nursing students to choose geriatric nursing as a career path is crucial for addressing the current and projected shortages in the geriatric nursing workforce. The complex relationships among geriatric nursing competence, attitudes toward older people, and geriatric nursing career motivation represent critical areas of study in the face of global demographic shifts [9, 26]. Theory of planned behavior [27] offers a robust theoretical framework for understanding these dynamics, which posits that attitude (e.g., attitudes toward older people) and perceived behavioral control (e.g., geriatric nursing competence) are key determinants of behavioral intentions (e.g., geriatric nursing career motivation). As our understanding of how these elements interact becomes clearer, new avenues for addressing challenges in the development of geriatric nursing workforce have opened. While previous studies have examined attitudes and competence as independent predictors of geriatric nursing career motivation [28], little is known about whether geriatric nursing competence mediates the relationship between attitudes toward older people and geriatric nursing career motivation, particularly in the Chinese context.

This study aimed to explore the interrelationships among attitudes toward older people, geriatric nursing competence, and motivation to pursue careers in geriatric nursing among nursing students, and whether geriatric nursing competence played a role in the relationship between attitudes toward older people and geriatric nursing career motivation. By examining these variables, this study contributes to the growing body of knowledge in geriatric nursing and provide insights that can inform future educational and policy initiatives in this crucial field. Therefore, the following hypotheses were proposed:

### Hypothesis 1

Nursing students with positive attitudes toward older people are likely to have high levels of geriatric nursing competence;

### Hypothesis 2

Nursing students with higher levels of geriatric nursing competence are more motivated to pursue a geriatric nursing career;

### Hypothesis 3

Geriatric nursing competence mediates the relationship between attitudes toward older people and geriatric nursing career motivation.

## Method

### Design and participants

A cross-sectional survey was conducted to collect data for this descriptive correlational study. The eligibility criteria for inclusion were as follows: (1) at least 18 years of age, (2) currently enrolled full-time in a four-year undergraduate nursing program, and (3) successfully completed the geriatric nursing course; and (4) provided informed consent to participate in the study. Students who unable to complete the survey during the investigation period due to taking a leave of absence from school was excluded. As the geriatric nursing course at this university is offered as an elective during the sophomore year, this study recruited students from the sophomore, junior, and senior years.

### Setting and procedures

Data are collected from a nursing school at a medical university, which is a large academic institution located in Wenzhou, an urban setting in Zhejiang Province, eastern China. A geriatric nursing course is offered as an elective course during the second year of the university, consisting of 25 h of teaching (23 h of theoretical teaching and 2 h of virtual simulation-based laboratory teaching). The course contents covered geriatric nursing theories and concepts, health assessment of older people, health promotion and disease prevention in older people, psychological care and mental health in older people, and assistance with activities of daily living and nursing care for older people etc. Data were collected between June and August 2023 via Wenjuanxing (www.wjx.cn), an online survey platform in China that allowed individual user accounts. Before conducting the study, the researchers consulted counselors of various grades, explained the purpose and procedures of the study, and obtained approval. A research assistant distributed an electronic questionnaire link through WeChat course groups, given WeChat’s popularity as a communication and social platform in China. The first page of the electronic questionnaire contained information on informed consent and research purpose, informing students of the voluntary nature of their participation and the confidentiality of their information. Students who voluntarily agreed to participate clicked on the link and completed the survey on a computer or smartphone. The questionnaire took approximately 10–15 min to complete and was configured with a forced-response feature, requiring participants to complete all items before submission. Therefore, there were no missing data, and no imputation or deletion procedures were necessary. In total, 676 questionnaires were administered. After excluding questionnaires completed in less than 10 min and those showing uniform answers, 529 valid questionnaires were retained for analysis, attaining an effective response rate of 78.25%.

### Measures

A structured questionnaire was used to collect data, including the following four subsections: a demographic questionnaire, the KAOP scale, the Geriatric Nursing Career Motivation questionnaire and the Geriatric Nursing Competence Scale for Clinical Nurses.

### Demographic information

The demographic information questionnaire designed by the researchers was used to collect data on sex, age, place of residence, and previous geriatric exposure of participants, which referred to participants’ previous experience in caring for older people, either at home (e.g., caring for older family members) or in hospital, clinical, or long-term care settings.

### The Chinese version of Kogan’s attitudes toward older people scale

The KAOP scale was used to measure attitudes toward older people. This scale was developed by Kogan [18] and translated by Yen [29]. It is a reliable tool for assessing students’ attitudes toward older people, and the Chinese version has been widely used and validated in China [16, 30]. The 34 items on the scale are divided into two dimensions: prejudice (negative items 1–17) and appreciation (positive items 18–34). The answers to individual items use seven-point Likert scales ranging from 1 (strongly disagree) to 7 (strongly agree); the positive scoring items 18–34 are counted as 1, 2, 3, 5, 6, and 7 points, respectively, with a score of 4 assigned in the rare case of failure to respond to an item, and the negative items 1–17 were the opposite. The total score of the Chinese version of the KAOP ranges from 34 to 238 points, with a higher score indicating a more positive attitudes toward older people. The original scale demonstrated Cronbach’s α for the overall Chinese version scale was 0.82, with the prejudice dimension at 0.83 and the appreciation dimension at 0.81 [29]. In this study, Cronbach’s α was 0.870 for the total scale, 0.941 for prejudice, and 0.920 for appreciation, which indicates good internal consistency reliability.

### Geriatric nursing career motivation questionnaire

The Geriatric Nursing Career Motivation Questionnaire was used to assess career motivation. The questionnaire was developed by Cheng in 2014 based on the expectancy-value theory and senses framework [31]. The 20 items in the questionnaire were divided into two sub-questionnaires: expectancy (6 items) and value (14 items). The value questionnaire includes four dimensions: interest (3 items), practicality (3 items), importance (5 items), and cost (3 items). The answers to individual items used a five-point Likert scale ranging from 1 (strongly disagree) to 5 (strongly agree), and the cost items were reverse-scored. The total score on the Geriatric Nursing Career Motivation questionnaire ranges from 20 to 100 points, the expectancy questionnaire score ranges from 6 to 30 points, and the value questionnaire score ranges from 14 to 70 points, with higher scores indicating that nursing students attribute a higher value to the geriatric nursing career and have higher expectations of themselves in this field. The original scale demonstrated Cronbach’s α was 0.83 for expectancy and 0.87 for value [31]. In this study, the Cronbach’s α for the expectancy and value questionnaires were 0.937 and 0.909, respectively, indicating excellent reliability.

### The geriatric nursing competence scale for clinical nurses

The Geriatric Nursing Competence Scale for Clinical Nurses was used to measure geriatric nursing competence that undergraduate nursing students believe they already possess and should possess in the future as clinical nurses. The scale was developed in 2019 based on the theoretical framework of a technology-oriented geriatric nursing behavioral competence assessment model [32]. This 43-item scale measures geriatric nursing competence in three domains: professional literacy competence (items 1–6: law and ethics; items 7–9: critical thinking; items 10–11: specialized knowledge), professional practice competence (items 12–22: clinical practice; items 23–25: safety management; items 26–30: communication and consultation), and professional development competence (items 31–32: quality management; items 33–38: leadership and education; items 39–40: professional learning; items 41–43: scientific research innovation). The answers to the individual items use five-point Likert scales ranging from 0 (strongly disagree) to 4 (strongly agree). The total score on the Geriatric Nursing Competence Scale ranges from 0 to 172 points, with higher scores indicating higher geriatric nursing competence. The original scale demonstrated Cronbach’s α for the overall scale was 0.978, with the three dimensions ranging from 0.912 to 0.966 [32]. In this study, Cronbach’s α was 0.983 for the total scale, 0.925 for professional literacy competence, 0.964 for professional practice competence and 0.973 for professional development competence, indicating excellent reliability.

### Statistical analysis

Statistical analyses were performed using SPSS version 26.0 and AMOS version 23.0 (both from IBM Corp., Armonk, NY, USA). A *p*-value of < 0.05 was considered significant.

### Analytical strategy

The analytical approach comprised three stages. First, preliminary analyses included descriptive statistics, common method bias assessment, and bivariate correlations among variables. Second, SEM was used to test the hypothesized mediation model. Third, sensitivity analysis incorporated gender, residence, and previous geriatric exposure as control variables to verify the robustness of the mediation relationships.

### Preliminary and descriptive analyses

For descriptive data, frequencies and percentages were used to summarize categorical variables (e.g., gender and residence), whereas means and standard deviations (M ± SD) were employed to characterize normally distributed continuous variables. Common method bias was assessed using Harman’s single-factor test to evaluate whether a single factor accounted for the majority of the variance among the study variables [33]. Bivariate Pearson correlations were calculated using the observed total scores to examine the unadjusted correlations among geriatric nursing competence, attitudes toward older people and geriatric nursing career motivation among undergraduate nursing students. This approach allows for providing overall picture of the associations between variables.

### Testing of hypotheses via SEM

Additionally, SEM was used to investigate the mediating role of geriatric nursing competence, via a model using latent variables constructed from multiple observed indicators, in the associations between attitudes toward older people and undergraduate nursing students’ geriatric nursing career motivation. This latent variable approach separates measurement error to obtain more accurate estimates of structural relationships and mediation effects. The measurement model specified three latent variables and their indicators. Geriatric nursing competence was indicated by professional literacy competence, professional practice competence, and professional development competence. Attitude towards older people was indicated by KAOP- (negative attitudes) and KAOP+ (positive attitudes). Geriatric nursing career motivation was indicated by expectancy and value. The covariates included gender, residence, and previous geriatric exposure. A two-step structural equation modeling approach was used to test the hypotheses. First, using a measurement model to assess the scale’s reliability and validity. Composite reliability (CR) was used to test internal consistency, CR values greater than 0.7 were accepted [34]. Average variance extracted (AVE) was used to test convergent validity, the AVE should be greater than 0.5 [34]. And the Fornell and Larcker’s criterion was used to test discriminant validity, the square root of a construct’s AVE should exceed its correlations with all other constructs in the model [34]. Second, using a structural model for path analyses. To evaluate the model’s goodness of fit, several indices were employed: the ratio of chi-square to degrees of freedom (χ2/df), root mean square error of approximation (RMSEA), standardized root mean square residual (SRMR), comparative fit index (CFI), the Tucker-Lewis index (TLI), and incremental fit index (IFI). The model was deemed to have adequate fit if it met the following cutoff criteria: χ2/df < 3; RMSEA < 0.06; SRMR < 0.08; and CFI, TLI, and IFI all exceeding 0.95 [35]. During the model refinement process, the modification indices (MI) and estimated parameter changes were used to improve the model fit. According to the covariance between variables, a step-by-step modified model was fit, and this iterative process continued until an adequate fit was achieved [36]. The direct and mediating effects were constructed using the maximum likelihood estimation method, and the bias-corrected bootstrap method was employed for testing. Specifically, 5000 bootstrap samples were generated to estimate 95% confidence intervals (CIs) to assess whether each hypothesized mediator accounted for the association between dependent and independent variables. Significance was determined by checking whether the interval bounds (Boot LLCI and Boot ULCI) include zero, effects with CIs excluding zero were considered statistically significant.

### Sensitivity analysis

Gender, place of residence, and previous geriatric exposure were incorporated into the SEM as control variables in sensitivity analyses. These covariates were specified as exogenous observed variables predicting all three endogenous latent variables to examine whether the hypothesized mediation relationships remained robust after controlling for these control variables.

### Ethical considerations

This study was reviewed and approved by the Institutional Review Board of Wenzhou Medical University (Approval number: 2023027). All the participants provided informed consent before participating in the study. The study was conducted in accordance with the Declaration of Helsinki and relevant ethical guidelines for research on human subjects.

## Results

### Data distribution of the variables

Results showed that the absolute values of skewness for all aforementioned variables across all profiles were below 3, and the absolute values of kurtosis were below 10 [37]. These results met the criteria for normality, supporting the use of parametric tests in the subsequent analyses.

### Demographic characteristics

A total of 529 nursing students completed the questionnaire, comprising 448 (84.7%) females and 81 (15.3%) males, with a mean age of 20.60 ± 0.90 years. The majority of the participants were sophomores (*n* = 292, 55.2%) and juniors (*n* = 235, 44.4%), were from rural areas (*n* = 358, 67.7%), and did not have experience in caring for older people (*n* = 418, 79%). The general characteristics of the participants are presented in Table 1.

**Table 1:** Demographic characteristics (*N* = 529)

Variable	Mean ± SD, (*N*)	%
**Age**	20.60 ± 0.90	/
**Sex**		
Male	81	15.3
Female	448	84.7
**Academic year**		
Sophomore year	292	55.2
Junior year	235	44.4
Senior year	2	0.40
**Place of residence**		
Rural	358	67.7
Urban	171	32.3
**Previous geriatric exposure**		
Yes	111	21
No	418	79

### Analysis of descriptive statistics

Results showed that the average scores for KAOP-, KAOP+, and the total KAOP were 78.79 (SD = 14.62), 71.3 (SD = 10.85), and 150.10 (SD = 16.07), respectively; professional literacy competence, professional practice competence, professional development competence and geriatric nursing competence were 35.99 (SD = 7.70), 58.41 (SD = 15.26), 37.69 (SD = 11.22), and 132.09 (SD = 32.44), respectively; and expectancy, value and geriatric nursing career motivation were 21.12 (SD = 4.11), 48.42 (SD = 6.90), and 69.54 (SD = 10.20), respectively.

### Common method bias assessment

Results revealed that 12 factors had eigenvalues greater than 1, with the first factor accounting for 30.63% of the total variance, which was below the critical threshold of 40% [38], indicating that serious common method bias was not a concern in this study.

### **Relationships among geriatric nursing competence**,** attitudes toward older people**,** and geriatric nursing career motivation**

Table 2 shows the results of the correlation analysis. Pearson’s correlation analysis showed that geriatric nursing competence was positively correlated with attitudes toward older people (*r* = 0.106, *p* < 0.05) and geriatric nursing career motivation (*r* = 0.416, *p* < 0.01), although geriatric nursing competence was non-significantly correlated with negative attitudes (*r*=-0.059, *p* > 0.05). Notably, attitudes toward older people were significantly positively correlated with geriatric nursing career motivation (*r* = 0.375, *p* < 0.01).

**Table 2:** Description statistics and correlations between geriatric nursing competence, attitude towards older people and geriatric nursing career motivation (*N* = 529)

Variables		Mean	SD	1	2	3	4	5	6	7	8	9	10
Geriatric nursing competence	1. Professional literacy competence	35.99	7.70	1									
	2. Professional practice competence	58.41	15.26	0.857**	1								
	3. Professional development competence	37.69	11.22	0.751**	0.887**	1							
	4. Geriatric nursing competence	132.09	32.44	0.900**	0.980**	0.941**	1						
Attitudes toward older people	5. KAOP-	78.79	14.62	0.001	-0.045	-0.108*	-0.059	1					
	6. KAOP+	71.31	10.85	0.230**	0.223**	0.221**	0.236**	-0.230**	1				
	7. Total KAOP	150.10	16.07	0.156**	0.110*	0.051	0.106*	0.754**	0.465**	1			
Geriatric nursing career motivation	8. Expectancy	21.12	4.11	0.415**	0.447**	0.449**	0.464**	-0.117**	0.506**	0.235**	1		
	9. Value	48.42	6.90	0.335**	0.320**	0.312**	0.338**	0.064	0.528**	0.415**	0.695**	1	
	10. Career motivation	69.54	10.20	0.394**	0.397**	0.392**	0.416**	-0.004	0.561**	0.375**	0.874**	0.957**	1

Note: KAOP, attitude towards old people; KAOP-, negative attitude towards old people; KAOP+, positive attitude towards old people. Geriatric nursing competence scale ranges 0-172 (higher = better), KAOP scale ranges 34–238 (higher = better), Gerontological nursing career motivation scale ranges 20–100 (higher = better)

*p < 0.05, **p < 0.01

### Measurement model assessment

The results of confirmatory factor analysis confirmed the construct validity of the measurement model. Table 3 provides information about the convergent validity and composite reliability. All constructs demonstrate satisfactory convergent validity, with AVE values exceeding the 0.5 threshold. Moreover, geriatric nursing competence and geriatric nursing career motivation demonstrate acceptable internal consistency with CR values. Table 4 provides information about the discriminant validity. The diagonal elements represent the square root of AVE for each construct, while all off-diagonal elements are lower than their corresponding diagonal values, thus confirming discriminant validity.

**Table 3:** Convergent validity and composite reliability of measurement model

Constructs	CR	AVE
Geriatric nursing competence	0.941	0.842
Attitudes toward older people	0.696	0.621
Geriatric nursing career motivation	0.822	0.698

CR: composite reliability; AVE: average variance extracted

**Table 4:** Discriminant validity of measurement model

	GNC	KOAP	GNCM
Geriatric nursing competence (GNC)	0.917		
Attitudes toward older people (KOAP)	0.208	0.788	
Geriatric nursing career motivation (GNCM)	0.474	0.559	0.835

### Mediating effect of geriatric nursing competence between attitudes toward older people and geriatric nursing career motivation

**Fig. 1:**
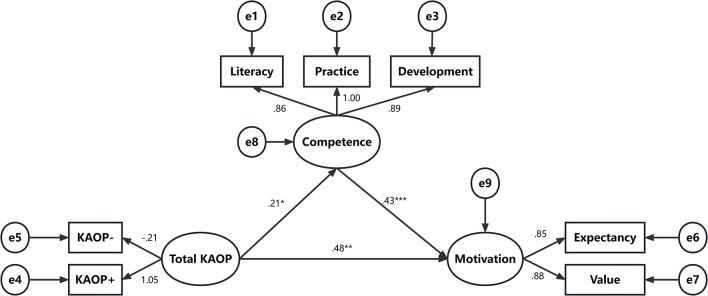
SEM of geriatric nursing competence, attitude towards old people and geriatric nursing career motivation (*N* = 529). Note: KAOP: attitude towards old people; KAOP-: negative attitude towards old people; KAOP+: positive attitude towards old people; Literacy: professional literacy competence; Practice: professional practice competence; Development: professional development competence; Competence: geriatric nursing competence; Motivation: geriatric nursing career motivation. e1 to e9: unobserved variables. *p < 0.05, **p < 0.01, ***p < 0.001

Based on the results of the correlation analysis, we hypothesized that geriatric nursing competence would mediate the relationship between attitudes toward older people and geriatric nursing career motivation. SEM was used to test the model, with attitudes toward older people as the independent variable, geriatric nursing career motivation as the dependent variable, and geriatric nursing competence as the mediating variable. The initial model fit was χ²/df = 7.505, CFI = 0.968, TLI = 0.940, IFI = 0.969, SRMR = 0.050, and RMSEA = 0.111, indicated poor model fit. The model was further refined by incorporating parameter MIs until achieving an acceptable mode fit. After three modifications, according to the model fit indicators cutoff criteria, this model showed acceptable goodness-of-fit indices, with χ²/df = 2.613, CFI = 0.994, TLI = 0.985, IFI = 0.994, SRMR = 0.0197, and RMSEA = 0.055. A bootstrap bias-corrected estimator was used to further test the model. Tables 5 and 6, and Fig. 1 show that the standardized path coefficients support that attitudes toward older people were associated with geriatric nursing competence (*β* = 0.215, SE = 0.050, *p* = 0.013). Thus, Hypothesis 1 was supported. In addition, geriatric nursing competence was associated with geriatric nursing career motivation (*β* = 0.430, SE = 0.030, *p* < 0.001), which supported Hypothesis 2. Furthermore, attitudes toward older people had a direct effect on geriatric nursing career motivation (*β* = 0.476, SE = 0.056, *p* = 0.009). Notably, the SEM path coefficient (*β* = 0.476) was substantially stronger than the observed correlation (*r* = 0.106), reflecting the correction for measurement error in the latent variable approach. The 95% bias-corrected CIs did not include zero for any of the paths. The indirect effect of attitudes toward older people on geriatric nursing career motivation via geriatric nursing competence was 0.028 (95% CI [0.009, 0.050]), and the direct effect of attitudes toward older people on geriatric nursing career motivation was 0.146 (95% CI: [0.028, 0.296]), indicating that the relationship between attitudes toward older people and geriatric nursing career motivation was partly mediated by geriatric nursing competence. The indirect effect of the path (Total KAOP → geriatric nursing competence → geriatric nursing career motivation) was 0.028 (bootstrap 95% CI [0.009, 0.050]), accounting for 16.10% of the total effect. Thus, Hypothesis 3 was supported.

**Table 5:** Model path coefficient test (*N* = 529)

Path	Nonstandardized coefficient	standardized coefficient	SE	CR(T)	95% CI		*p*
					Lower	Upper	
Total KAOP → Geriatric nursing competence	0.125	0.215	0.050	2.482	0.029	0.250	0.013
Geriatric nursing competence →Geriatric nursing career motivation	0.226	0.430	0.030	7.459	0.168	0.307	< 0.001
Total KAOP →Geriatric nursing career motivation	0.146	0.476	0.056	2.625	0.028	0.296	0.009

Note: KAOP, attitude towards old people

**Table 6:** Indirect, direct, and total effect analysis of attitude towards old people and geriatric nursing career motivation (*N* = 529)

Path	Effect Size	Bootstrap 95%CI			Effect proportion (%)
		Lower	Upper		
**Indirect effects**					
Total KAOP → Geriatric nursing competence→ Geriatric nursing career motivation	0.028	0.009		0.050	16.091
**Direct effect**					
Total KAOP → Geriatric nursing career motivation	0.146	0.028		0.296	83.908
**Total effect**					
Total KAOP → Geriatric nursing career motivation	0.174	0.037		0.334	100.000

Note: KAOP, attitude towards old people

### Sensitivity analysis

**Fig. 2:**
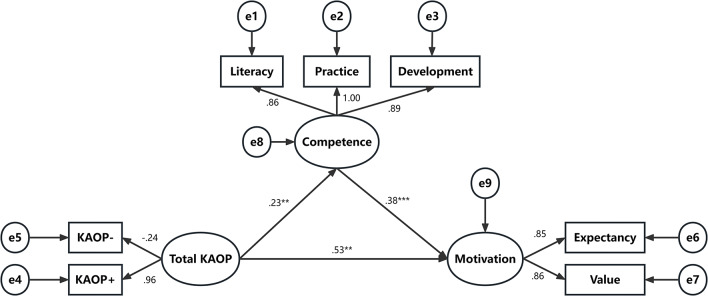
SEM of geriatric nursing competence, attitude towards old people and geriatric nursing career motivation (Control variables: gender, place of residence, and previous geriatric exposure) (*N* = 529). Note: KAOP: attitude towards old people; KAOP-: negative attitude towards old people; KAOP+: positive attitude towards old people; Literacy: professional literacy competence; Practice: professional practice competence; Development: professional development competence; Competence: geriatric nursing competence; Motivation: geriatric nursing career motivation. e1 to e9: unobserved variables. *p < 0.05, **p < 0.01, ***p < 0.001

Control variables, including gender, place of residence, and previous geriatric exposure, were incorporated into the model to re-examine the path coefficients and assess the robustness of the findings (Fig. 2). The model demonstrated satisfactory fit indices (χ²/df = 2.304, CFI = 0.986, TLI = 0.975, IFI = 0.986, SRMR = 0.0409, and RMSEA = 0.050). The path effects of the key variables were as follows: attitudes toward older people on geriatric nursing career motivation (*β* = 0.525, SE = 0.053, *p* = 0.001), attitudes toward older people on geriatric care competence (*β* = 0.225, SE = 0.049, *p* = 0.003), and geriatric care competence on geriatric nursing career motivation (*β* = 0.376, SE = 0.029, *p* < 0.001). The explained variance of the three paths increases significantly, thereby corroborating the previous analytical results and confirming the stability of the proposed model.

## Discussion

This study aimed to investigate the relationships among geriatric nursing competence, attitudes toward older people, and geriatric nursing career motivation among nursing students. Results revealed that students reported moderate levels of attitudes toward older people and geriatric nursing career motivation, with a moderate positive correlation observed between these two variables. Our findings on attitudes toward older people align with those from diverse cultural contexts, including Turkey, Africa [39], Nepal [40], the United States, and Canada [41]. However, the results concerning geriatric nursing career motivation have diverged across studies [42]. This may be attributed to societal attitudes toward aging, undergraduate curricula, clinical experiences, and healthcare working conditions [43], which collectively contribute to the challenge of attracting new nurses in geriatric nursing. However, positive experiences can counteract these negative effects. In our study, 21% of the students had experience caring for older people. Research demonstrates that such exposure, whether through clinical practice, family interactions, or structured learning, is associated with enhanced geriatric nursing competence and stronger career motivation [9, 16, 44].

Consistent with hypothesis 1, our results also revealed that attitudes towards older people were associated with geriatric nursing competence. This aligns with previous research that has demonstrated similar correlations between positive attitudes and both willingness to work with older people and geriatric competence [8], though evidence remains limited. Geriatric nursing competence is crucial as it directly influences patient health outcomes and the quality and effectiveness of nursing care delivery [45]. Prior study also pointed out that nurses’ attitudes toward older people should be actively changed in order to provide quality care to older people and improve their geriatric nursing competence. This may be attributed to positive attitudes promoting the acquisition of aging-related knowledge and its integration into nursing care [46]. This relationship underscores the importance of cultivating positive attitudes toward older people in nursing education to potentially increase students’ geriatric nursing competence [47].

Supporting hypothesis 2, the results confirmed that geriatric nursing competence is associated with geriatric nursing career motivation. This also aligns with previous studies, which shows that greater geriatric nursing competence improves perceived value, self-efficacy, and increases the likelihood of choosing geriatric nursing career [42, 48, 49]. According to expectancy-value theory [50], improved competence strengthens career expectations by increasing students’ confidence in their ability to perform effectively in geriatric settings and their appreciation of intrinsic value, personal meaning, and practical value, thus strengthening their career expectations and subjective valuation of geriatric nursing [51]. The mechanisms underlying this relationship may be explained by several interconnected factors. Geriatric nursing competence enables student to understand the individuality and unique healthcare needs of older people [52], and this enhanced understanding is positively linked to interest in the profession [53]. Moreover, mastering geriatric nursing competence decreases students’ resistance to the field and enables more active clinical interactions with older people by increasing in professional confidence and self-efficacy [43], which in turn strengthens their interest in a geriatric nursing career. Additionally, students with greater geriatric nursing competence gain awareness of career development opportunities and challenges in this field [54]. This cognition broadens their career horizons and increases the likelihood of choosing geriatric nursing as their career direction [55, 56].

This study revealed that attitudes toward older people had a significant direct effect on nursing students’ motivations to choose a career in geriatric nursing, which was in line with previous studies [9, 16, 43, 57, 58]. Students who view older people favorably may be more inclined to view geriatric nursing as a rewarding and meaningful career path [59]. However, studies in Sri Lanka and Australia found that despite holding positive attitudes toward older people and recognizing the career’s value, students still demonstrated limited interest and motivation in pursuing geriatric nursing careers [60, 61]. This discrepancy may be explained by cultural differences, which may shape attitudes toward aging and geriatric nursing career motivation. Cultural values like filial piety, which uphold respect for the elderly, may explain why nursing students from Asian collectivist societies often hold more positive attitudes toward older people [54] and consequently greater motivation to pursue geriatric nursing careers.

This study also found that the relationship between attitudes toward older people and geriatric nursing career motivation was partially mediated by geriatric nursing competence, supporting hypothesis 3. This suggests that positive attitudes toward older people may motivate students to acquire geriatric nursing knowledge and skills, thereby increasing their competence, which in turn enhances their willingness to work in this field [15]. However, the effect size was modest (*β* = 0.028), accounting for only 16.09% of the total effect, which warrants careful interpretation. Although statistically significant, the indirect effect was small in magnitude, thus offering limited explanatory power for the relationship between attitudes toward older people and geriatric nursing career motivation. It is likely that additional unmeasured mediators or moderators not included in the current model, such as gratitude [16] and students’ expectations for digital technology used in aging care services [62], may have a more significant effect on the relationship between attitudes toward older people and geriatric nursing career motivation. Future studies should explore, identify and examine such mechanisms to better explain their relationships.

### Practical implications

These findings have profound practical implications for nursing education and healthcare policies. First, the study reveals the small but significant mediating role of geriatric nursing competence, suggesting that enhancing students’ professional competence may contribute to their motivation to pursue geriatric nursing careers. Nursing programs could therefore provide comprehensive geriatric nursing education and clinical experiences to equip students with relevant knowledge and skills. Innovative teaching methods such as reflective learning (e.g., problem-based learning (PBL) [63]) and simulation training (e.g., role-playing workshops [64]) may enhance students’ geriatric nursing competence. Second, the findings underscore the importance of cultivating positive attitudes toward older people among nursing students to enhance their motivation for geriatric nursing careers. Curricula should integrate experiential learning (e.g., empathy skills training [65]), which can deeply understand the circumstances and needs of older people and simulation and technology enhanced learning and simulation training (e.g., artificial intelligence-based learning activities [66]), which enables students to authentically interact with older people in safe and controlled virtual environments, thereby fostering positive attitudes toward older people. Finally, the increasing demand for geriatric nursing professionals in an aging population calls for strategic policy initiatives to support the development of the geriatric nursing care workforce. Comprehensive incentive systems, including tuition subsidies, improved salary structures, and holistic support for students specializing in geriatric nursing [67] should be implemented. By addressing these issues, educational institutions and policymakers can help bridge the supply-demand gap in the geriatric nursing workforce, ultimately improving the quality of care for older people.

### Strengths

This study is the first to investigate the mediating role of geriatric nursing competence in the relationship between nursing students’ attitudes toward older people and their career motivation in the Chinese cultural context. These findings advance our theoretical understanding of the psychological mechanisms underlying career choice in geriatric nursing. Moreover, results have direct practical implications for nursing education, providing empirical evidence supporting targeted interventions, such as curriculum reform, simulation-based teaching, and reflective learning in clinical training, to cultivate more positive attitudes and strengthen professional competence among nursing students, ultimately attracting more qualified individuals to geriatric care.

### Limitations

This study has several limitations that might lead to overestimation of the observed relationships. First, our sample was drawn exclusively from one medical university in Wenzhou and consisted predominantly of pre-clinical sophomore and junior students. This limited representativeness may introduce a potential selection bias and restrict the generalizability of our findings. As curricula [68], different cultural attitudes toward aging [54] and clinical experience may substantially influence students’ perceptions and motivations across regions and institutions. Future studies should enroll more diverse samples from multiple centers to control for these factors. Second, since the Geriatric Nursing Competence Scale for Clinical Nurses was originally designed for clinical nurses rather than students, respondents may have rated their expected rather than actual competence. Future studies should employ questionnaires specifically designed for nursing students to accurately measure their abilities. Third, the cross-sectional study design limit to explore causal relationships between variables; therefore, future studies should use longitudinal designs. Additionally, despite controlling for several confounding variables, the study may have been subject to residual confounding because of unmeasured variables. A potential example is course exposure, as enrollment in geriatric nursing courses can be associated with more positive attitudes, higher self-rated competence, and greater career motivation. Fourth, reliance on self-reported measurements introduces potential response biases. Students may express more than they actually hold due to social desirability bias. Future studies should adopt more objective measures to evaluate nursing students’ genuine thoughts. Fifth, although Harman’s single-factor test indicated that common method bias may not be a severe issue, this single statistical test may not fully capture all potential sources of method variance. Future research should employ multi-method approaches (e.g., combining self-reports with objective measures or peer ratings) to address potential common method variance more rigorously and strengthen causal inferences. Finally, since all participants had completed the elective geriatric nursing course, we could not compare them with students who had not taken the course. Future research should employ comparison groups or longitudinal designs to distinguish educational effects from selection effects.

## Conclusion

This study revealed positive correlations among nursing students’ geriatric nursing competence, attitudes toward older people, and geriatric nursing career motivation. Notably, attitudes toward older people were directly associated with geriatric nursing career motivation, with geriatric nursing competence playing a small but significant mediating role. These findings suggest the importance of fostering positive attitudes and enhancing professional competence in nursing education. To attract more students to geriatric nursing, a comprehensive approach that includes providing rich practical experiences, cultivating empathy, eliminating stereotypes, and strengthening professional knowledge may help nurture a more competent and compassionate geriatric nursing workforce. Such efforts may contribute to addressing the challenges faced by the aging population and improving their quality of life.

## Data Availability

The datasets generated and analyzed during the current study are not publicly available due to confidentiality agreements with study participants, but are available from the corresponding author on reasonable request.

## References

[1] World Health Organization. Ageing and health 2024 [Available from: https://www.who.int/news-room/fact-sheets/detail/ageing-and-health

[2] YuWa Population Research. Chinese population forecast report 2023 [Available from: https://file.c-ctrip.com/files/6/yuwa/0R70l12000ap4aa8z4B12.pdf

[3] Chai X, Cheng C, Mei J, Fan X. Student nurses’ career motivation toward gerontological nursing: A longitudinal study. Nurse Educ Today. 2019;76:165–71. 10.1016/j.nedt.2019.01.028. 30784845 10.1016/j.nedt.2019.01.028

[4] World Health Organization. Global strategy and action plan on ageing and health 2017 [Available from: https://www.who.int/publications/i/item/9789241513500

[5] Dann T. Global elderly care in crisis. Lancet. 2014;383(9921):927. 24629279 10.1016/S0140-6736(14)60463-3

[6] Feng Z. Global convergence: Aging and long-term care policy challenges in the developing world. J Aging Soc Policy. 2019;31(4):291–7. 31154942 10.1080/08959420.2019.1626205

[7] Harris J. Geriatric trends facing nursing with the growing aging. Crit Care Nurs Clin. 2019;31(2):211–24. 10.1016/j.cnc.2019.02.007 31047094

[8] LiuYE, Norman IJ, While AE. Nurses’ attitudes towards older people: a systematic review. Int J Nurs Stud. 2013;50(9):1271–82. 10.1016/j.ijnurstu.2012.11.021. 10.1016/j.ijnurstu.2012.11.021 23265870

[9] Wang Y, Yang J, Wang L, Liu J, Xin T, Gao L, Liu Y. Nursing students’ motivation for choosing a career in geriatric nursing: A cross-sectional study. Educ Gerontol. 2024;50(8):673–82. 10.1080/03601277.2024.2328892.

[10] Koskinen S, Burke E, Fatkulina N, Fuster P, Löyttyniemi E, Salminen L, et al. Graduating nurse students’ interest in older people nursing—A cross-sectional survey in six European countries. Int J Older People Nurs. 2022;17(3):e12446. 10.1111/opn.12446. 10.1111/opn.12446 PMC9285737 35080809

[11] Cheng M, Cheng C, Tian Y, Fan X. Student nurses’ motivation to choose gerontological nursing as a career in China: a survey study. Nurse Educ Today. 2015;35(7):843–8. 10.1016/j.nedt.2015.03.001. 10.1016/j.nedt.2015.03.001 25792382

[12] Haron Y, Levy S, Albagli M, Rotstein R, Riba S. Why do nursing students not want to work in geriatric care? A national questionnaire survey. Int J Nurs Stud. 2013;50(11):1558–65. 10.1016/j.ijnurstu.2013.03.012. 10.1016/j.ijnurstu.2013.03.012 23664143

[13] Dai F, Liu Y, Ju M, Yang Y. Nursing students’ willingness to work in geriatric care: an integrative review. Nurs Open. 2021;8(5):2061–77. 10.1002/nop2.726. 10.1002/nop2.726 PMC8363346 34388864

[14] Garbrah W, Kankkunen P, Välimäki T. Gerontological nurse teachers’ abilities and influence on students’ willingness in older people nursing: A cross-sectional, correlational survey. Nurse Eduction Today. 2020;90:104461. 10.1016/j.nedt.2020.104461. 10.1016/j.nedt.2020.104461 32408244

[15] Birimoglu Okuyan C, Bilgili N, Mutlu A. Factors affecting nursing students’ intention to work as a geriatric nurse with older adults in Turkey: A cross-sectional study. Nurse Educ Today. 2020;95:104563. 10.1016/j.nedt.2020.104563. 33032122 10.1016/j.nedt.2020.104563

[16] Zhang S, Liu YH, Zhang HF, Meng LN, Liu PX. Determinants of undergraduate nursing students’ care willingness towards the elderly in China: Attitudes, gratitude and knowledge. Nurse Educ Today. 2016;43:28–33. 10.1016/j.nedt.2016.04.021. 27286941 10.1016/j.nedt.2016.04.021

[17] Jeyasingam N, McLean L, Mitchell L, Wand APF. Attitudes to ageing amongst health care professionals: a qualitative systematic review. Eur Geriatr Med. 2023;14(5):889–908. 10.1007/s41999-023-00841-7. 37553540 10.1007/s41999-023-00841-7 PMC10587319

[18] Kogan N. Attitudes toward old people: the development of a scale and an examination of correlates. J Abnorm Soc Psychol. 1961;62:44–54. 10.1037/h0048053. 13757539 10.1037/h0048053

[19] American Association of Colleges of Nursing & Hartford Institute for Geriatric Nursing. Recommended baccalaureate competencies and curricular guidelines for the nursing care of older adults 2010 [Available from: https://www.aacnnursing.org/Portals/0/PDFs/CCNE/AACN-Gero-Competencies-2010.pdf

[20] Nunnelee J, Tanner EI, Cotton A, Harris M, Alderman J, Hassler L, et al. NGNA: position paper on essential gerontological nursing education in registered nursing and continuing education programs. Geriatric nursing (New York, NY). 2015;36(3):239–41. 10.1016/j.gerinurse.2015.04.011. 10.1016/j.gerinurse.2015.04.011 26244180

[21] Sakamoto RR. Influence of RAP experiences on nursing students’ career choices in aging: A qualitative exploration. Nurse Educ Today. 2022;109:105218. 10.1016/j.nedt.2021.105218. 34799195 10.1016/j.nedt.2021.105218

[22] Eldardery NE-S, Ebied EME-S, Mohammed Y, Khodary K, Mohammed B, Mohammed S, et al. Exploring internship nursing students’ perception of gerontological nursing competencies: A descriptive study at Cairo University Hospitals. J Integr Nurs. 2023;5(4):235–42. 10.4103/jin.jin_91_23.

[23] Lee H, Gu M, Sok S. The Effects of Nurses’ Perception of the Older Adults and Work Stress on Nursing Competency of Nurses Who Care for Older Adult Patients at General Hospital. Int J Environ Res Public Health. 2023;20(3):2095. 36767460 10.3390/ijerph20032095 PMC9916333

[24] Jemal K, Hailu D, Mekonnen M, Tesfa B, Bekele K, Kinati T. The importance of compassion and respectful care for the health workforce: a mixed-methods study. Z Gesundh Wiss. 2023;31(2):167–78. 10.1007/s10389-021-01495-0. 10.1007/s10389-021-01495-0 PMC7951938 33728258

[25] Cybulska AM, Żołnowska MA, Schneider-Matyka D, Nowak M, Starczewska M, Grochans S, Cymbaluk-Płoska A. Analysis of nurses’ attitudes toward patient death. Int J Environ Res Public Health. 2022;19(20):13119. 36293697 10.3390/ijerph192013119 PMC9602489

[26] Castellano-Rioja E, Botella-Navas M, López-Hernández L, Martínez-Arnau FM, Pérez-Ros P. Caring for the elderly enhances positive attitudes better than knowledge in nursing students. Med (Kaunas). 2022;58(9). 10.3390/medicina58091201. 10.3390/medicina58091201 PMC9506512 36143876

[27] Ajzen I. The theory of planned behavior: Frequently asked questions. Hum Behav Emerg Technol. 2020;2(4):314–24.

[28] Guo Y, Yang L, Zhu L, Wan Y, Zhang S, Zhang J. Willingness and associated factors of working with older people among undergraduate nursing students in China: a cross-sectional study. BMC Nurs. 2021;20(1):113. 10.1186/s12912-021-00639-7. 34182971 10.1186/s12912-021-00639-7 PMC8237413

[29] Yen C-H, Liao W-C, Chen Y-R, Kao M-C, Lee M-C, Wang C-C. A Chinese version of Kogan’s Attitude toward Older People Scale: Reliability and validity assessment. Int J Nurs Stud. 2009;46(1):38–44. 10.1016/j.ijnurstu.2008.05.004. 10.1016/j.ijnurstu.2008.05.004 18701105

[30] Yao G, Luo Y, Zhao Z, Zhu B, Gao M. The moderating role of empathy profiles in the relationship between knowledge about aging and attitudes toward older adults among nursing students. Front Psychol. 2021;12:713271. 34733203 10.3389/fpsyg.2021.713271 PMC8558622

[31] Cheng M, Fan X, Tian Y, Cheng C. Development of Gerontological Nursing Career Motivation Questionnaire for nursing students. J Nurs Sci. 2014;29:53–6.

[32] Wei. L, Liang H, Lu Y, Mo M, Zhang X, Cai M. [The development and psychometric test of Geriatric Nursing Competence Scale for Clinical Nurses]. Chin J Nurs. 2019;54(9):1378–84.

[33] Podsakoff PM, MacKenzie SB, Lee J-Y, Podsakoff NP. Common method biases in behavioral research: a critical review of the literature and recommended remedies. J Appl Psychol. 2003;88(5):879. 14516251 10.1037/0021-9010.88.5.879

[34] Cheung GW, Cooper-Thomas HD, Lau RS, Wang LC. Reporting reliability, convergent and discriminant validity with structural equation modeling: A review and best-practice recommendations. Asia Pac J Manage. 2024;41(2):745–83.

[35] Hu L-t, Bentler PM. Cutoff criteria for fit indexes in covariance structure analysis: Conventional criteria versus new alternatives. Struct Equ Model. 1999;6:1–55.

[36] Whittaker TA. Using the Modification Index and Standardized Expected Parameter Change for Model Modification. J Experimental Educ. 2012;80(1):26–44. 10.1080/00220973.2010.531299.

[37] Byrne BM, van de Vijver FJR. Testing for Measurement and Structural Equivalence in Large-Scale Cross-Cultural Studies: Addressing the Issue of Nonequivalence. Int J Test. 2010;10(2):107–32. 10.1080/15305051003637306.

[38] Hayes A. Introduction to mediation, moderation, and conditional process analysis. 2013. 335-7 p.

[39] Faronbi JO, Adebowale O, Faronbi GO, Musa OO, Ayamolowo SJ. Perception knowledge and attitude of nursing students towards the care of older patients. Int J Afr Nurs Sci. 2017;7:37–42.

[40] Ghimire S, Shrestha N, Callahan KE, Nath D, Baral BK, Lekhak N, Singh DR. Undergraduate nursing students’ knowledge of aging, attitudes toward and perceptions of working with older adults in Kathmandu Nepal. Int J Nurs Sci. 2019;6(2):204–10. 31406893 10.1016/j.ijnss.2019.03.003 PMC6608660

[41] Hovey S, Dyck MJ, Reese C, Kim M. Nursing students’ attitudes toward persons who are aged: An integrative review. Nurse Educ Today. 2017;49:145–52. 27930921 10.1016/j.nedt.2016.11.018

[42] Ben Natan M, Danino S, Freundlich N, Barda A, Yosef RM. Intention of nursing students to work in geriatrics. Res Gerontol Nurs. 2015;8(3):140–7. 25707032 10.3928/19404921-20150219-03

[43] Neville C, Dickie R, Goetz S. What’s Stopping a Career in Gerontological Nursing? Literature Review. J Gerontol Nurs. 2014;40(1):18–27. 10.3928/00989134-20131126-02. 24296568 10.3928/00989134-20131126-02

[44] Keeping-Burke L, McCloskey R, Donovan C, Yetman L, Goudreau A. Nursing students’ experiences with clinical placement in residential aged care facilities: a systematic review of qualitative evidence. JBI Evid synthesis. 2020;18(5):986–1018. 10.11124/JBISRIR-D-19-00122 32813353

[45] Chen H, Pu L, He S, Hu X, Chen Q, Huang Z, Cheng L. Status and associated factors of gerontological nurse specialists’ core competency: a national cross-sectional study. BMC Geriatr. 2023;23(1):450. 10.1186/s12877-023-04153-0. 37479983 10.1186/s12877-023-04153-0 PMC10362742

[46] Hammad BM, Salameh B, Eqtait FA, Maysa K, Fashafsheh IH, Ayed AJ, et al. Nursing students’ knowledge, attitudes, and behaviors toward aging and ageism in Palestine. BMC Geriatr. 2025;25(1):296. 10.1186/s12877-025-05946-1. 40307722 10.1186/s12877-025-05946-1 PMC12042619

[47] Venables H, Wells Y, Fetherstonhaugh D, Wallace H. Factors associated with nursing students’ attitudes toward older people: A scoping review. Gerontol Geriatr Educ. 2023;44(1):131–50. 10.1080/02701960.2021.2012466. 34927567 10.1080/02701960.2021.2012466

[48] Fu JX, Huang LL, Li XH, Zhao H, Li R. Association between ageing knowledge and willingness to care for older adults among nursing students in China: the mediating role of attitude towards older adults. Gerontol Geriatr Educ. 2024;45(3):444–57. 10.1080/02701960.2023.2227874. 37356028 10.1080/02701960.2023.2227874

[49] Potter G, Clarke T, Hackett S, Little M. Nursing students and geriatric care: The influence of specific knowledge on evolving values, attitudes, and actions. Nurse Educ Pract. 2013;13(5):449–53. 23465846 10.1016/j.nepr.2013.02.007

[50] Du C, Han R, Li C. An exploration of the construction of china’s eldercare service talent team from the expectancy theory perspective. Open J Bus Manage. 2017;5(3):501–13.

[51] Tomita R. The relationship between general self-efficacy and nursing practice competence for second‐year nurses: Empirical quantitative research. Nurs Open. 2024;11(7):e2233. 38961662 10.1002/nop2.2233 PMC11222662

[52] Miron AM, Schmidt BJ, Schlueter A, Patterson M, O’Connell S. Improving nursing students’ perspective taking, perceptions of humanness, and attitudes toward older adults. Gerontol Geriatr Educ. 2021;42(4):564–77. 10.1080/02701960.2019.1621864. 31130108 10.1080/02701960.2019.1621864

[53] Chi MJ, Shyu ML, Wang SY, Chuang HC, Chuang YH. Nursing students’ willingness to care for older adults in Taiwan. J Nurs scholarship: official publication Sigma Theta Tau Int Honor Soc Nurs. 2016;48(2):172–8. 10.1111/jnu.12197. 10.1111/jnu.12197 26824721

[54] Garbrah W, Välimäki T, Palovaara M, Kankkunen P. Nursing curriculums may hinder a career in gerontological nursing: An integrative review. Int J Older People Nurs. 2017;12(3):e12152. 10.1111/opn.12152 28397376

[55] Gould ON, MacLennan A, Dupuis-Blanchard S. Career preferences of nursing students. Can J Aging. 2012;31(4):471–82. 10.1017/s0714980812000359. 23084578 10.1017/S0714980812000359

[56] Koehler AR, Davies S, Smith LR, Hooks T, Schanke H, Loeffler A, et al. Impact of a stand-alone course in gerontological nursing on undergraduate nursing students’ perceptions of working with older adults: A Quasi-experimental study. Nurse Eduction Today. 2016;46:17–23. 10.1016/j.nedt.2016.06.015. 10.1016/j.nedt.2016.06.015 27475123

[57] Che CC, Chong MC, Hairi NN. What influences student nurses’ intention to work with older people? A cross-sectional study. Int J Nurs Stud. 2018;85:61–7. 10.1016/j.ijnurstu.2018.05.007. 29852374 10.1016/j.ijnurstu.2018.05.007

[58] Krumova P. Motivation and attitude of nurse students for working with the elderly and old people. Knowl Int J. 2024;65(4):437–41.

[59] King BJ, Roberts TJ, Bowers BJ. Nursing student attitudes toward and preferences for working with older adults. Gerontol Geriatr Educ. 2013;34(3):272–91. 10.1080/02701960.2012.718012. 23383875 10.1080/02701960.2012.718012 PMC3659195

[60] Neville C. A cross-sectional view of Australian undergraduate nurses’ perceptions of working with older people. Collegian. 2016;23(3):285–92.

[61] Rathnayake S, Athukorala Y, Siop S. Attitudes toward and willingness to work with older people among undergraduate nursing students in a public university in Sri Lanka: A cross sectional study. Nurse Educ Today. 2016;36:439–44. 26507448 10.1016/j.nedt.2015.10.007

[62] Feng Y, Wang Y, Liang C, Lu L, Xie C. The effect of digitalization on the career intentions of nursing students: A cross-sectional study. Nurse Educ Pract. 2023;71:103726. 37499535 10.1016/j.nepr.2023.103726

[63] Cheng Y, Sun S, Hu Y, Wang J, Chen W, Miao Y, Wang H. Effects of different geriatric nursing teaching methods on nursing students’ knowledge and attitude: Systematic review and network meta-analysis. PLoS ONE. 2024;19(5):e0300618. 38820259 10.1371/journal.pone.0300618 PMC11142439

[64] Benko E, Peršolja M. Nursing students’ views of the impact of geriatric role-play workshops on professional competencies: survey. BMC Nurs. 2023;22(1):203. 37316872 10.1186/s12912-023-01373-y PMC10265884

[65] Başer G, Hisar F. Effect of interventions for health care students to develop positive attitude toward the elderly: a meta-analysis study. Geriatr Gerontol Int. 2024;24(12):1370–9. 10.1111/ggi.15012. 10.1111/ggi.15012 PMC11628901 39500564

[66] White A, Maguire MB, Brown A, Keen D. Impact of artificial intelligence on nursing students’ attitudes toward older adults: A pre/post-study. Nurs Rep. 2024;14(2):1129–35. 38804418 10.3390/nursrep14020085 PMC11130905

[67] Resnick B, Young HM, Fick DM, Kagan SH. Making care for older people the choice of nurses today, tomorrow, and forever. Volume 46. New York, NY: Geriatric Nursing; 2022. p. A1. 10.1016/j.gerinurse.2022.04.014 PMC9122780 35606212

[68] Ho M-H, Lee JJ, Joo JY, Bail K, Liu MF, Traynor V. Determinants of the intention to work in aged care: a cross-sectional study to assess gerontological nursing competencies among undergraduate nursing students. BMC Nurs. 2023;22(1):448. 38031123 10.1186/s12912-023-01613-1 PMC10685655

